# Catechins: Therapeutic Perspectives in COVID-19-Associated Acute Kidney Injury

**DOI:** 10.3390/molecules26195951

**Published:** 2021-09-30

**Authors:** Lúcio Ricardo Leite Diniz, Hatem A. Elshabrawy, Marilia Trindade de Santana Souza, Allana Brunna Sucupira Duarte, Sabarno Datta, Damião Pergentino de Sousa

**Affiliations:** 1 College of Nordeste da Bahia, Coronel João Sá 48590-000, BA, Brazil; luciodiniz@yahoo.com.br; 2 Department of Molecular and Cellular Biology, College of Osteopathic Medicine, Sam Houston State University, Conroe, TX 77304, USA; hatem.elshabrawy@shsu.edu; 3 Department of Pharmacy, Federal University of Sergipe, São Cristóvão 49100-000, SE, Brazil; biomari@hotmail.com; 4 Department of Pharmaceutical Sciences, Federal University of Paraíba, João Pessoa 58051-970, PB, Brazil; allanabrunna@gmail.com; 5 College of Osteopathic Medicine, Sam Houston State University, Conroe, TX 77304, USA; sxd071@shsu.edu

**Keywords:** SARS-CoV-2, coronavirus, renal disease, nephrotoxicity, reno-protective effect, green tea, epigallocatechin-3-gallate (EGCG), flavonoids, natural products, medicinal plant

## Abstract

Data obtained from several intensive care units around the world have provided substantial evidence of the strong association between impairment of the renal function and in-hospital deaths of critically ill COVID-19 patients, especially those with comorbidities and requiring renal replacement therapy (RRT). Acute kidney injury (AKI) is a common renal disorder of various etiologies characterized by a sudden and sustained decrease of renal function. Studies have shown that 5–46% of COVID-19 patients develop AKI during hospital stay, and the mortality of those patients may reach up to 100% depending on various factors, such as organ failures and RRT requirement. Catechins are natural products that have multiple pharmacological activities, including anti-coronavirus and reno-protective activities against kidney injury induced by nephrotoxic agents, obstructive nephropathies and AKI accompanying metabolic and cardiovascular disorders. Therefore, in this review, we discuss the anti-SARS-CoV-2 and reno-protective effects of catechins from a mechanistic perspective. We believe that catechins may serve as promising therapeutics in COVID-19-associated AKI due to their well-recognized anti-SARS-CoV-2, and antioxidant and anti-inflammatory properties that mediate their reno-protective activities.

## 1. Introduction

Coronaviruses (CoVs) are positive single-stranded (+ss) RNA viruses that are known to infect several mammalian and avian species [1,2]. Seven human CoVs (HCoVs) have been reported as the causative agents of respiratory diseases, of which only three are associated with severe respiratory illnesses with high fatality, whereas others cause mild symptoms [3,4]. Severe Acute Respiratory Syndrome-CoV (SARS-CoV) was first discovered following a disease outbreak that occurred in China in 2002–2003 [5]. Middle East Respiratory Syndrome-CoV (MERS-CoV) was then discovered in 2012 after reports of severe respiratory disease cases in Saudi Arabia [6]. In December 2019, SARS-CoV-2 was identified as the novel causative agent of Coronavirus Disease 2019 (COVID-19) that originated in Wuhan, China, and later became a pandemic, resulting in millions of deaths worldwide [4]. SARS-CoV-2 is transmitted primarily via respiratory droplets, after which it infects and replicates in lung epithelial cells, causing respiratory symptoms observed in COVID-19 patients [7].

The SARS-CoV-2 uses its receptor binding domain (RBD), within the S1 domain of spike (S) protein on the viral surface, for binding to the angiotensin converting enzyme 2 (ACE2) receptor and infecting lung cells [8]. After binding to ACE2, S protein is processed by cell membrane-bound serine protease TMPRSS2 or cathepsin L in late endosomes into S1 and S2 subunits [9,10]. A fusion peptide in the S2 domain then triggers the fusion of the viral envelope with cellular membranes, releasing the viral RNA into the cytoplasm [9]. Similar to SARS-CoV, which shares 80% genomic sequence identity with SARS-CoV-2, there is a cap at the 5′ end of the SARS-CoV-2 genome and a poly-A tail at the 3′ end [11,12]. Once the SARS-CoV-2 genome is in the cytoplasm, the 5′ end is translated into two polyproteins, pp1a and pp1ab [12]. These two polyproteins are processed by two proteases, main protease (Mpro or 3CLpro) and papain-like protease (PLpro), which are part of polyproteins. Processing of pp1a and pp1ab produces 16 nonstructural proteins (NSP1–16) with different roles in viral replication, including RdRp (NSP12) and others which have enzymatic activity, such as NSP15 (endoribonuclease) and NSP13 (helicase) [12]. The rest of the genome is translated into structural and nonstructural proteins. The structural proteins, S, membrane (M), envelope (E) and nucleocapsid (N) proteins assemble with the viral RNA, following genome replication, to form new viral particles which are released from infected cells to initiate infection of neighboring healthy cells [12].

COVID-19 patients have shown an unpredictable and variable clinical outcome that ranges from asymptomatic presentation to multiple organ failure and death [13,14,15]. Although pulmonary complications have been the main clinical presentation of COVID-19, there is increasing evidence that acute kidney injury (AKI) is strongly associated with the in-hospital high mortality of COVID-19 patients, especially those with underlying comorbidities and/or who require renal replacement therapy (RRT) [16,17,18]. AKI can lead to serious complications, such as hydro-electrolytic disturbance, alterations in blood pressure control, impairment of acid–base homeostasis, rise of serum concentration of toxic metabolites and increased risk of drug overdose [19,20]. Moreover, AKI contributes to the production of diverse inflammatory mediators and vasoactive agents that play key roles in pathological mechanisms of several diseases [21].

Although at the beginning of the COVID-19 pandemic renal involvement in severe COVID-19 was insignificant, clinical reports have shown that AKI is associated with the severity of the disease and in-hospital deaths of COVID-19 patients [16,22,23]. The incidence of AKI in hospitalized COVID-19 patients can vary from 5% to 46%, which most likely require RRT. It has been estimated that approximately 20% of COVID-19 patients, admitted to intensive care units (ICUs), would require RRT on average 15 days after disease onset [24,25,26]. The prolonged hospitalization and poor prognosis of COVID-19 patients with AKI who require RRT are not observed, at least in the same magnitude, in COVID-19 patients with other commodities or renal disease. For example, SARS-CoV-2-infected patients on peritoneal dialysis or with chronic kidney disease (CKD) not requiring RRT showed no significant difference in incidence and mortality compared to the general population, while COVID-19 patients with dialysis-dependent CKD showed greater risk of in-hospital deaths [27,28]. Therefore, patients on dialysis with earlier-stage renal disease requiring RRT have become a vulnerable group to COVID-19-related morbidity and mortality, and it is extremely necessary to find an effective treatment for impairment of renal functions and damages promoted by SARV-CoV-2 infection.

In this context, reno-protective compounds that showed therapeutic activity in different experimental models of AKI and antiviral activity against SARS-CoV-2 would be ideal in COVID-19-associated AKI [29].

Catechins are natural polyphenolic compounds found in a wide variety of fruits, vegetables and plant-based food and beverages. Green tea extract is a recognized rich dietary source of catechins, containing a substantial amount of catechin, (-)-epicatechin (EC), (-)-epicatechin-3-gallate (ECG), (-)-epigallocatechin (EGC) and (-)-epigallocatechin-3-gallate (EGCG) [30,31]. A broad range of pharmacological activities have been reported for catechins, including antiviral and nephroprotective activities, which are strongly associated with their anti-inflammatory and antioxidant properties [32,33,34,35,36,37].

In the present study, we review the anti-SARS-CoV-2 activity of catechins, safety and effectiveness of catechins in AKI induced by diverse nephrotoxic stimuli, and provide evidence that catechins could be used as antiviral and reno-protective agents to prevent COVID-19-induced AKI.

## 2. Materials and Methods

We performed a literature search of catechins, coronaviruses and acute kidney injury. The literature search, performed in the PubMed database, included studies published in English until March 2021 and used the following keywords: catechins and COVID-19, catechins and coronaviruses, catechins and SARS-CoV-2, catechins and acute kidney injury, catechins and acute renal failure, catechins and SARS-COV-2-induced acute kidney injury, catechins and SARS-COV-2-induced acute renal injury and catechins and COVID-19-induced acute renal injury. We selected studies which investigated the anti-SARS-CoV-2 activities of catechins as well as the reno-protective effects of catechins and isomers in experimental or clinical AKI in accordance with KDIGO stages 1, 2 or 3 definitions. Studies on the reno-protective activities of crude extracts or beverages as well as combinations of catechins with other bioactive drugs were not included. Reported data of reno-protective effects attributed to catechins assessed by in vitro assays or performed in experimental models of chronic renal injury were not selected.

## 3. Catechins: General Pharmacological Properties

Catechins are natural polyphenolic compounds, belonging to the flavanols group of flavonoids, found in a wide variety of fruits, vegetables and plant-based food and beverages, such as fresh tea leaves, red wines, black grapes, cocoa beans, apricots and others [31,34]. Green tea extract is a recognized rich dietary source of catechins, containing a substantial amount of catechin, (-)-epicatechin (EC), (-)-epicatechin-3-gallate (ECG), (-)-epigallocatechin (EGC) and (-)-epigallocatechin-3-gallate (EGCG). EGCG is the most active and abundant polyphenol in green tea, accounting for approximately 50% of green tea polyphenols [30,31,32,33,34,35,36,37,38].

Pharmacokinetic studies have shown that, after oral administration, catechins and their metabolites are absorbed by the gastrointestinal tract and may undergo three types of biotransformation in humans: methylation, glucuronidation and sulfation in the liver and intestinal tissues [39,40,41]. Following absorption in the small intestine, the peak plasma concentrations (nmol/L) of EC, EGC and EGCG might vary from 1.5 to 2.5 h after intraduodenal administration of 20 and 30 mg/kg BW catechins, and return to baseline values between 2.5 and 4 h after administration, respectively [39]. The catechin metabolites are conjugated in the liver and excreted in the urine. The urinary excretion levels of catechins vary from 0% to 28.5% in human volunteers [40,41]. The bioavailability of catechins depend on several factors, such as enterohepatic recirculation, used dose, interaction with food components and flavan-3-ol stereochemistry. Different catechins were ranked as (-)-EC > (+)-EC = (+)-catechin > (-)-catechin on the basis of plasma concentrations and urinary excretion of the aglycones [38,39,40,41,42,43]. Recently, Andreu-Fernandez et al. [44] reported that the oral administration of EGCG in the form of green tea extract, in a single dose of 250 mg after overnight fasting, resulted in the highest peak concentrations (Cmax), area under the curve (AUC) values of 0–360 and average concentrations (Cav) both in men (5.95 ng/mL/kg, 3.86 ± 4.11 µg/mL/kg/6 h, 2.96 ng/mL/kg) and women (6.66 ng/mL/kg, 3.33 ± 1.08 µg/mL/kg/6 h, 3.66 ng/mL). Moreover, the study reported that t1/2 after oral ECGC administration were 192 ± 66 and 133 ± 28 min in men and women, respectively. A Mediterranean diet breakfast was shown to reduce the bioavailability of EGCG (more than 100% in males and 30% in females) [44].

Over the last few years, studies have demonstrated a broad range of pharmacological activities of catechins, including neuroprotective, anticarcinogenic, antihypertensive, antibacterial, antiviral and anti-inflammatory activities [45,46,47]. The majority of therapeutical indications attributed to catechins are strongly associated with their anti-inflammatory and antioxidant properties [48,49,50,51,52,53,54]. Catechins possess direct antioxidant effects, through free radical scavenging and metal ion chelation, as well as indirect antioxidant effects by induction of antioxidant enzymes, inhibition of pro-oxidant enzymes and production of the phase II detoxification enzymes [50,51]. Interestingly, studies have shown that catechins may produce a dose-dependent pro-oxidant effect, due to activation of the nuclear factor-erythroid factor 2-related factor 2 (Nrf2) pathway, leading to production of ROS [52,53].

**Figure 1:**
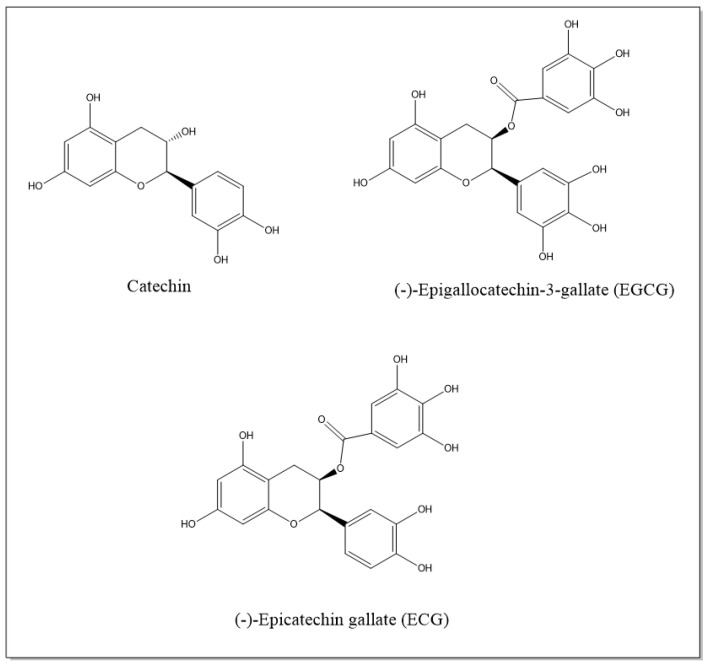
Chemical structure of reno-protective catechins.

Studies have provided evidence that pure catechins or plants with high concentrations of catechins exert anti-inflammatory effects in inflammatory diseases [48,49]. Mechanistic studies revealed that catechins decrease the production of pro-inflammatory cytokines, such as TNF-α, IL-1β, IL-6 and INF-γ, reduce the expression of adhesion molecules, inhibit infiltration and proliferation of immune cells and decrease the activity of inflammatory-related enzymes, such as iNOS and COX-2. Catechins’ therapeutic effects are mediated by impairing multiple inflammation-related and oxidative stress-related pathways that involve JNK/NF-κB, MAPKs, Nrf2 and STAT1/3 transcription factors [49,54]. Figure 1 illustrates the catechins discussed in this study.

## 4. Anti-SARS-CoV-2 Activity of Catechins

**Figure 2:**
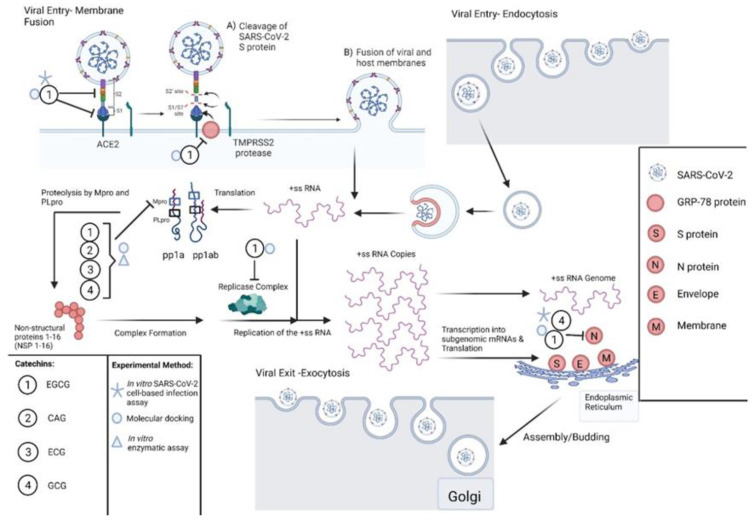
Catechins exert anti-SARS-CoV-2 activities by targeting different steps of the SARS-CoV-2 lifecycle. Catechins, such as EGCG, bind to SARS-CoV-2 S protein and inhibit its binding to the ACE2 receptor. EGCG also binds to GRP-78, which potentially blocks its binding to S protein, which may inhibit viral entry. EGCG, ECG, CAG and GCG inhibit Mpro of SARS-CoV-2, which blocks viral replication. Molecular docking studies have shown that EGCG binds to RNA-dependent RNA polymerase and other proteins of the replicase complex (NSP6 and NSP15) which may block viral replication. Furthermore, EGCG and GCG bind to and inhibit association of N protein with the RNA genome blocking viral assembly. This figure was created with BioRender.com (accessed on 25 July 2021).

Given the urgent need to develop effective antiviral drugs against SARS-CoV-2, many research groups have tested several drug candidates, including plant-derived compounds, for anti-SARS-CoV-2 activity. Polyphenols, such as catechins, have been well-known for their antiviral activity against several RNA viruses, including coronaviruses [55,56,57,58,59,60]. A study has shown that epigallocatechin-3-gallate (EGCG) inhibited the SARS-CoV main protease (Mpro or 3CLpro) with an IC_50_ of 73 μM [59]. Another study has shown that increasing the concentration of (-)-catechin gallate (CAG) gallate potently inhibited SARS-CoV N protein and its association with RNA, with an IC_50_ of 0.05 μg/mL [61]. Based on their effectiveness against SARS-CoV, several studies have tested catechins for anti-SARS-CoV-2 activities (Figure 2). Molecular docking studies have shown that EGCG, epicatechin-3-gallate (ECG) and CAG bind strongly to SARS-CoV-2-Mpro’s amino acid residues, His41 and Cys145, that are important for the enzymes’ catalytic activity [62,63,64]. The Mpro-catechins complexes were found to be of high stability, which indicates that they can be further developed into potent Mpro inhibitors and SARS-CoV-2 antivirals. The high-affinity bindings of ECG and EGCG to Mpro were confirmed by an in vitro study which showed that ECG and EGCG inhibited recombinant Mpro activity with IC_50_ values of 5.21 ± 0.5 and 7.51 ± 0.21, respectively [65]. Furthermore, CAG and (-)-gallocatechin-3-gallate (GCG) inhibited Mpro with IC_50_ values of 2.98 ± 0.21 and 6.38 ± 0.5, respectively. EGCG was also reported by another group as an inhibitor of Mpro [66].

A molecular docking study of 18 plant constituents to SARS-CoV-2 proteins showed that EGCG bound with higher affinity than the antiviral drugs chloroquine and remdesivir to all tested SARS-CoV-2 protein targets, including Mpro, S protein, S2 subunit of the S protein, RBD-ACE2 complex and NSP15 endoribonuclease [67]. The previous study was in agreement with another molecular docking study which showed high binding affinity of EGCG and catechin to SARS-CoV-2 proteins such as Mpro, RNA-dependent RNA polymerase, PLpro, RBD of S protein, NSP6, N protein, ACE2 receptor and ACE2 receptor bound to RBD [68,69,70,71]. The binding of EGCG to SARS-CoV-2-S protein was further confirmed by a molecular docking study which showed high-affinity binding to S protein [72], and another study which showed high-affinity binding not only to the S protein of the wild-type strain but to the UK mutant strain S protein [73]. The ability of EGCG to target S protein and its potential to impair its binding to the ACE2 receptor indicates that it could be further developed as an entry inhibitor for SARS-CoV-2. Furthermore, all docking studies provide evidence that EGCG and other catechins target multiple SARS-CoV-2 viral proteins, which make catechins potentially effective antivirals against SARS-CoV-2 and emerging variants since it would not be feasible for the virus to mutate all viral targets to avoid inhibition. Glucose-Regulated Protein-78 (GRP78) is an ER protein which plays a major role in the unfolded protein response (UPR), ensuring proper folding of proteins and reducing the amounts of unfolded proteins [74]. Studies have shown that GRP-78 binds to SARS-CoV-2-S protein, and this interaction is critical for viral entry [75]. Moreover, EGCG was shown to bind to and inhibit GRP-78 and thus could prevent its binding to SARS-CoV-2-S protein [74,76]. These findings suggest that EGCG could be investigated as a potential viral entry inhibitor for SARS-CoV-2 by disrupting SARS-CoV-2-S protein–GRP-78 binding.

The previous research findings were complemented by in vitro SARS-CoV-2 infection assays using Vero E6 cells, which showed that the catechin mixture, extracted from green tea, inhibited viral replication and reduced viral titer in a dose-dependent manner [77]. Another study identified EGCG as an entry inhibitor not only for SARS-CoV-2 but also for SARS-CoV and MERS-CoV [78]. Furthermore, 1 mM of EGCG reduced the infectivity of SARS-CoV-2 by binding to S protein and impairing its interaction with the ACE2 receptor [79]. The previous study findings are consistent with molecular docking studies which showed high-affinity binding of EGCG to SARS-CoV-2-S protein [67]. Moreover, EGCG was reported as a NSP15 inhibitor and effectively blocked SARS-CoV-2 viral replication, with an IC_50_ = 0.2 M [80]. In addition to EGCG, GCG was also reported as an inhibitor for SARS-CoV-2 replication by targeting N protein, with an IC_50_ = 44.4 M and a selectivity index (SI) of 3.5 [81]. Interestingly, a mixture of catechins consisting of 32.8% EGCG, 15.2% ECG, 13.2% EC, 10.8% EGC, 10.4% GC and 4.4% catechin impaired SARS-CoV-2 replication in Vero E6 cells [82]. Moreover, the same study demonstrated the in vivo efficacy of the mixture in inducing cell-mediated immunity by increasing the frequency of CD8+ T cells and mitigating lipopolysaccharide-induced cytokine storm in mice. The in vivo activity of catechins implies that it can be used not only to block viral replication but to alleviate symptoms, due to SARS-COV-2-induced cytokine storm, and boost immunity against SARS-CoV-2.

## 5. Reno-Protective Effect of Catechins in the Acute Kidney Injury

**Figure 3:**
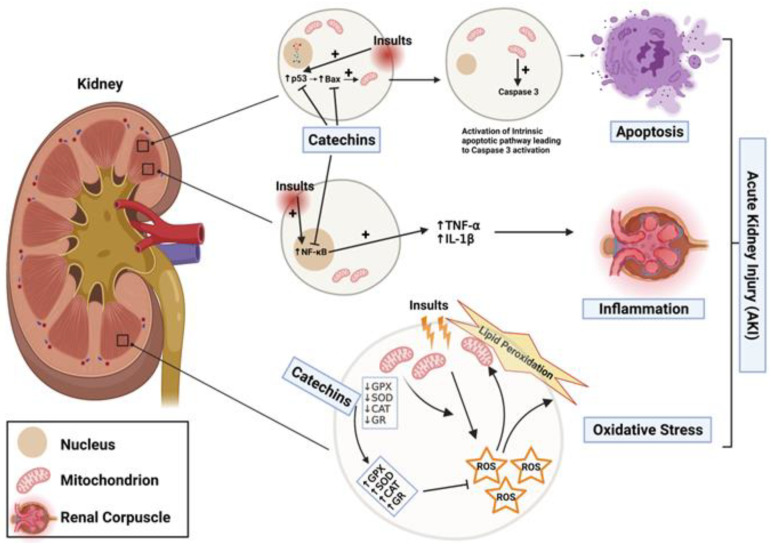
Catechins protect against AKI through different mechanisms. Catechins exert their reno-protective effects by inhibiting apoptosis (inhibition of p53 induction and Bax expression), reducing inflammation by decreasing accumulation of NF-kB in the nucleus, which lowers production of pro-inflammatory cytokines (TNF- α and IL-1 β), and attenuating oxidative stress and lipid peroxidation by restoring antioxidant enzyme activities, which detoxify reactive oxygen species (ROS). This figure was created with BioRender.com (accessed on 1 August 2021).

The reno-protective effects of catechins (Figure 3) have been investigated in experimental models of AKI over the past few years [32,33]. As illustrated in Table 1, several studies have shown that catechins and their isomers significantly reduced the impairment of renal function and structural damages caused by nephrotoxic drugs, obstructive nephropathy and AKI accompanying metabolic and cardiovascular disorders [83,84,85,86,87,88,89].

Pretreatment of animals with catechin (40 mg/kg, p.o.), twice a day for 4 days, prevented deterioration of kidney function and preserved renal morphology following injection of 50% glycerol solution (8 mL/kg, i.m.) and ferric nitrilotriacetate (8 mg iron/kg, i.p.) [83,90]. In a traditional model of AKI caused by a nephrotoxic dose of gentamicin (100 mg/kg/day, i.p.), the treatment with catechin hydrate (50 mg/kg), once daily for 14 days, inhibited the significant increases of BUN and SCr and protected glomeruli and tubules against gentamicin-induced damage [91]. In a nephrotoxicity model induced by cisplatin, it has been shown that 15 mg/kg of EGCG significantly inhibited the elevation of BUN and SCr induced by cisplatin (7 mg/kg) in rats [84]. In another study, 100 mg/kg of EGCG reduced SCr and BUN as well as kidney structural damages such as tubular dilatation, cast formation, granulovaculoar degeneration and tubular cell necrosis induced by cisplatin (20 mg/kg, i.p.) [92]. Similar reno-protective effects were reported by i.p. pretreatment with 5 mg/kg of ECG for 10 days, which protected against renal dysfunction and tubular necrosis induced by cisplatin (8 mg/kg) [86]. Furthermore, Gao et al. [93] showed that IV administration of EGCG (10 and 20 mg/kg) was effective in protection against contrast-induced nephropathy (CIN). The prophylactic or therapeutic treatment with EGCG was effective in CIN, as determined by low SCr and BUN levels, and a reduction in renal damage [93]. Soussi et al. [88] also showed that 5 mg/kg of EGCG protected against ammonium metavanadate (AMV)-induced glomerular hypertrophy and tubular dilatation in male Wistar rats.

In obstructive nephropathy models of AKI, catechins significantly reduced renal structural and functional abnormalities, whereas in an ischemia/reperfusion (I/R) model of AKI, pretreatment with catechin (40 mg/kg, p.o.), twice daily for 4 days and 2 h prior to renal ischemia, markedly attenuated renal dysfunction and morphological alterations induced by I/R nephropathy [94]. Lv et al. showed that i.p. treatment with 50 mg/kg of EGCG protected the kidneys and prevented histological changes induced by clamping the left renal artery for 45 min, followed by 24 h reperfusion and contralateral nephrectomy in rats [95]. It was also found that i.p. administration of 5 mg/kg of EGCG for 14 days alleviated glomerular and tubular injury, inhibited renal tubulointerstitial fibrosis and reduced tubular cell apoptosis in a mouse model of unilateral ureteral obstruction (UUO) [96]. Moreover, i.p. pretreatment with 50 mg/kg of EGCG promoted a marked decrease of mRNA levels of TNF-α and the two markers of kidney damage, KIM-1 and NGAL, in the obstructed kidney of UUO Wistar rats. However, there was no significant effect of EGCG on renal blood flow, GFR, urine volume and urinary sodium excretion [86]. Considering the severity and the timeline of AKI onset induced by the UUO model, we believe that the use of higher doses of EGCG could have a more profound therapeutic effect [97,98].

AKI is also a common comorbidity in cardiovascular and metabolic disorders, such as hypertension and diabetes. It was observed that administration of 5 mg/kg of catechins in the drinking water for 12 weeks prevented the progression of kidney damage in streptozotocin-induced diabetic rats [32,33]. In addition, Funamoto et al. investigated the reno-protective effect of oral pretreatment with EGCG for 2 weeks before cardiopulmonary bypass (CPB) in diabetic rats. The study reported that 30 min of CPB induced renal damage and that pretreatment with EGCG attenuated tubular injury and reduced KIM-1 expression [85]. Recently, Luo et al. reported that the oral administration of 50 mg/kg of EGCG, twice daily for 6 weeks, decreased blood pressure, lowered the 24 h urine protein levels and creatinine clearance and reduced the severity of renal fibrosis in Dahl rats with salt-sensitive hypertension [99]. The previous findings suggest that ECGC protected the kidneys, leading to a decrease in blood pressure. Treatment with EGCG also decreased serum cystatin C levels (an early marker for acute kidney injury), and urinary N-acetyl-β-d-glucosaminidase, NAG (a tubular injury marker), in high-fat diet-induced kidney injury [97]. Since AKI is strongly associated with high mortality of COVID-19 patients with underlying comorbidities, including diabetes and hypertension [98,99], the reno-protective effects of catechins are very promising and should be investigated in COVID-19-induced AKI.

In general, the mechanisms underlying nephrotoxicity are: (a) kidney-specific mitochondrial oxidative stress, caused by altered activities of mitochondrial electron transport chain enzyme complexes and accompanied by impaired antioxidant defenses, (b) inflammation, as evidenced by elevated levels of inflammatory mediators, such as TNF-α, IL-1, increased activities of NF-κB and p53 induction, and (c) apoptotic cell death, as indicated by high caspase-3 activity and DNA fragmentation in diverse clinical and experimental studies of AKI [100,101]. Oxidative stress is an important risk factor for AKI, as evidenced by a decrease in antioxidant enzymes’ activities, an increase in lipid peroxidation and elevated levels of reactive oxygen species (ROS) and reactive nitrogen species (RNS) in AKI induced by nephrotoxic drugs and obstructive nephropathies [52,53,54,55]. In view of this, the improvement of renal function following catechins treatment is frequently accompanied by reduced levels of thiobarbituric acid reactive substances (TBARS, a marker of lipid peroxidation) and restoration of renal antioxidant enzymes. This indicates that catechins exert reno-protective effects, probably by radical scavenging and antioxidant activities [52,53,54]. It has been shown that (+)-catechin, catechin hydrate and ECGC attenuate lipid peroxidation and preserve activities of antioxidant enzymes in several AKI experimental models, including kidney injury induced by cisplatin, gentamicin, ammonium metavanadate, Fe-NTA, streptozotocin and unilateral urethra obstruction and renal ischemia/reperfusion models [32,33,83,88,89,91,94].

In line with previous antioxidant activities of catechins, a study has shown that treatment of mice with EGCG markedly attenuated cisplatin-induced mitochondrial oxidative/nitrative stress [84,87,92]. According to Zhou et al., the antioxidant activities of catechins might be partly via activation of the Nrf2 signaling pathway because ECG increased mRNA and protein levels of Nrf2 and γ-glutamylcysteine synthetase (γ-GCS) in obstructive AKI induced by renal ischemia and reperfusion [89]. Increased levels of heme oxygenase-1 (HO-1) were reported in contrast-induced nephropathy (CIN), while treatment with EGCG further increased HO-1 levels accompanied by an increase in Nrf2. Interestingly, the blockade of HO-1 with protoporphyrin IX zinc (II) (ZnPP) reversed the protective effect of EGCG on CIN. Its ability to increase the activity of antioxidant enzymes and to reduce inflammation and oxidative stress indicates that HO-1 is the upstream molecule that regulates the EGCG therapeutic effects [93]. Additionally, Funamoto et al. reported that reduced expression of KIM-1 following EGCG treatment was accompanied by lower production of 8-hydroxy-20-deoxyguanosine, indicating reduced oxidant stress [85].

The reno-protective effects of catechins are also due to their specific targeting of inflammatory and apoptotic pathways that are well-described in the genesis and progression of renal injuries. Pretreatment with EGCG promoted a significant decrease of the renal expression of proinflammatory cytokines TNF-α, IL-1β and IL-6 and reduced the numbers of macrophages and T cells infiltrating the renal tissue in salt-induced renal injury in Dahl salt-sensitive rats [99]. Moreover, EGCG decreased macrophage infiltration and inflammatory cytokine production, and attenuated renal interstitial fibrosis, in UUO animals, through regulation of NF-κB, Nrf2 and TGF-β/Smad signaling pathways [48,49,54,92,93]. Moreover, treatment with catechins reduced the expression of proapoptotic proteins Bax and caspase-3, increased the expression of the antiapoptotic protein, Bcl-2, and attenuated the activation of the MAPK pathway in renal tissues of rat models of AKI [94,95,96,97].

## 6. COVID-19 and Acute Kidney Injury (AKI)

AKI is defined according to RIFLE (Risk, Injury, Failure, Loss, End-Stage Kidney Disease), Acute Kidney Network (AKIN) and Kidney Disease Improving Global Outcomes (KDIGO). Briefly, RIFLE uses the Glomerular Filtration Rate (GFR) measurement based on levels of creatinine in serum (SCr) and urine, and urine output over 7 days. Risk, Injury and Failure stages were determined by increases in serum creatinine (SCr) ≥ 1.5-, 2- and 3-fold from a known baseline, respectively [102,103,104]. AKIN diagnosis of AKI is driven by observations of minor increases in SCr over a shorter period of time (48 h). AKIN stage 1 is defined as an increase in SCr by ≥0.3 mg/dL (≥26.4 μmol/L) over 48 h, whereas an absolute rise in SCr to ≥2- and 3-fold above baseline are criteria for diagnosis and classification of AKIN stages 2 and 3, respectively [19,105]. The current definition of AKI according to KDIGO is similar to AKIN, but the timeframe is extended from 48 h to 7 days. The decrease of urinary output to less than 0.5 mL kg^−1^ h^−1^ for 6 h was also similar to the RIFLE and AKIN definitions [19,106].

AKI has been shown to develop in COVID-19 patients 5 to 9 days after hospital admission and mostly in patients with severe COVID-19 disease [107,108]. In general, elevated levels of SCr and blood urea nitrogen (BUN) and reduced GFR were reported in COVID-19 patients following hospital admission, whereas an increase in SCr accompanied by proteinuria or hematuria was observed in 7–63% and 26.7% of cases, respectively [20,109,110,111]. Hypokalemia with increased kaliuresis were also observed in COVID-19 patients, which could be resulting from elevated angiotensin II levels, alterations of tubular reabsorption of potassium ions by drugs such as diuretics and/or SARS-CoV-2-induced diarrhea [112,113,114].

The postmortem histopathological analysis of renal tissues of patients who died of COVID-19 showed significant glomerular and tubular lesions [115,116]. Diffuse acute proximal tubular injury and detachment of podocytes containing numerous spherical particles typical of viral inclusion bodies were observed in these tissues [117,118,119]. In addition, diffuse erythrocyte aggregation and obstruction of glomerular and peritubular capillaries were evident. In a few cases, platelets, thrombi or fibrinoid necrosis were detected within the glomerular capillary loops [117,118]. All the previous data have suggested glomerular ischemia, endothelial cell injury and coagulation activation in COVID-19 patients [114]. Glomerular and tubular damages were also reported in other viral infections. Membranous glomerulopathy, glomerulosclerosis, membranoproliferative glomerulonephritis, interstitial nephritis and necrotizing tubulointerstitial nephritis are common clinical manifestations caused by HIV, HCV, HBV and adenoviral infections [120,121,122]. Histological examination demonstrated acute distal tubular necrosis and the presence of viral particles in epithelial cells, as well as in Bowman’s capsule, which differ significantly from pathological changes reported in bacterial infection [122,123,124].

The precise mechanisms of AKI development in COVID-19 patients are not yet fully understood. However, the mechanisms might be divided into specific and unspecific mechanisms [23,24,117]. Among the specific mechanisms are the direct invasion of renal parenchyma by SARS-CoV-2 and the imbalance of the renin-angiotensin-aldosterone system (RAAS) with generation of inflammatory mediators, oxidative stress and microthrombosis [125,126]. The viral invasion of renal tissue has been described as an important mechanism for the development of virus-associated kidney injury [126,127]. The detection of viral particles in urine and the absence of CD4+ and CD8+ T cells in kidney tissue for a long time after initial viral infection suggest that immune cells are not recruited to infected renal tissue as a viral strategy to establish persistent infection [128,129,130]. RAAS has also been shown to play a key role in renal dysfunctional and AKI [131]. RAAS is comprised of the classical axis that consists of angiotensin-converting enzyme (ACE), angiotensin II (Ang II) and the angiotensin type 1 receptor (AT1R), and the alternative axis, which is composed of ACE2, Ang 1–7 and the Mas receptor [132,133,134]. The two RAAS axes act as counter-regulatory systems. Increases of ACE expression and activity lead to intense production of Ang II, which by binding to AT1R results in vasoconstriction, inflammation and pro-proliferative effects [132,134]. The reno-protective effects of ACE inhibitors and AT1R blockers in diabetic nephropathy and renal ischemia have suggested that the classical RAS axis is involved in the pathogenesis and progression of nephropathies [135,136]. On the other hand, several studies have shown that Ang 1–7, via Mas receptor activation, were reno-protective by counteracting AT1R-mediated Ang II renal damage [133,134,137,138]. It has been suggested that SARS-CoV-2 promoted both inhibition of ACE2 activity and lysosomal degradation of membrane-bound ACE2. Downregulation of ACE2 in SARS-CoV-2 infection leads to accumulation of Ang II, resulting in increased inflammation, fibrotic and vasoconstrictor effects [139,140,141,142]. Recently, Yang et al. demonstrated that kidney injury molecule-1 (KIM1), a molecule dramatically upregulated upon kidney injury, binds with the receptor-binding domain (RBD) of SARS-CoV-2 and MERS-CoV, facilitating their attachment to the cell membrane, with the immunoglobulin variable Ig-like (Ig V) domain of KIM1, which in turn suggest KIM1 as a novel receptor for SARS-CoV-2 and other coronaviruses. According to the authors, KIM1 may mediate and exacerbate the renal infection of SARS-CoV-2 in a ‘vicious cycle’, and KIM1 could be further explored as a therapeutic target [140].

The unspecific mechanisms of COVID-19-induced AKI are similar to those described in kidney impairment induced by different etiologies, such as renal ischemia/reperfusion, nephrotoxic compounds and bacterial infections [26,141]. Glomerular and tubular damages are believed to occur secondary to ischemia with the redistribution of blood flow from renal medulla to the cortex, the deterioration of microcirculatory oxygenation and the generation of local inflammatory mediators, pro-fibrotic agents and reactive oxygen species (ROS) [142,143]. A strong cytokines storm, characterized by overproduction of type I (IFN-α/β) and type II IFNs (IFN-γ), IL-1β, IL-6, IL-2 and IL-4, followed by a sudden decrease of virus loads in the mesangial cells and vascular endothelial cells, was described following Duck Hepatitis A virus (DHAV) infection [120,121,122,123,144]. The potential role of cytokine storm in COVID-19-induced kidney damage has also been described [24,124]. In addition, immune reactions or immune complex deposition during viral infections may play an important role in severe coagulopathy, endothelial damage and increased vascular permeability, resulting in glomerular membrane proliferation [24,120,121,122,123,124].

## 7. Conclusions

Catechins are phytochemicals present in several natural foods and medicinal plants with high therapeutic potential against various pathologies, especially in inflammatory diseases [29,30,31,32,33,34,35]. According to studies discussed in this review, these natural products exhibit significant nephroprotective effects in blocking or attenuating renal dysfunctions and glomerulus/tubular lesions caused by varied nephrotoxic origins [86,87,88,89,90,91,92,93,94,95,96,97]. Even though more studies are needed to elucidate the mechanisms underlying nephroprotective effects of catechins, the renoprotection promoted by catechins appears to be strongly associated with their antioxidant, anti-inflammatory and anti-apoptotic activities into renal tissues [100,101]. Among the catechins discussed, ECGC is a very promising nephroprotective and anti-SARS-CoV-2 compound, especially due to the antiviral activities discussed earlier in this review [67,68,69]. Together, the experimental and clinical data in the present review support the indication of catechins as promising molecules in the treatment of COVID-19-associated AKI [83,84,85,86,87,88,89,90,91,92,93,94,95,96,97,98,99]. In addition, catechins can also be used as a prototype for the synthesis of more active analogs for the treatment of AKI.

## Figures and Tables

**Table 1:** Reno-protective effects of catechins in animal models of acute kidney injury (AKI).

Acute Kidney Injury Induced by Nephrotoxic Drugs			
Experimental Model	Reno-Protective Effect	Mechanism	Reference
Rhabdomyolysis-induced AKI (50% glycerol-8 mL/kg, i.m.)	Catechin (40 mg/kg) inhibited the increase of BUN and SCr	Reduced the lipid peroxidation and increased glutathione levels. Restored the activity of renal antioxidant enzymes (SOD, CAT and GR).	[90]
Fe-NTA-induced AKI (8 mg iron/kg, i.p.)	Catechin (40 mg/kg) inhibited the increase of BUN and SCr	Reduced the lipid peroxidation and increased glutathione levels. Restored the activities of renal antioxidant enzymes (SOD, CAT and GR).	[83]
Gentamicin-induced AKI (100 mg/kg/day, i.p, for 14 days)	Catechin hydrate (50 mg/kg) inhibited the increase of BUN and SCr	Restored levels of renal glutathione.	[91]
Cisplatin-induced AKI (7, 8 and 20 mg/kg, i.p.)	EGCG (50 mg/kg) inhibited the increase of BUN and SCr	Restored the Se, Zn and Cu ion concentration in renal tissue. Restored the activities of renal SOD, GPX and CAT. Reduced lipid peroxidation.	[84]
	EGCG (100 mg/kg) improved cisplatin-induced tubular dilatation, cast formation, granulovaculoar degeneration and tubular cell necrosis	Restored the activities of renal antioxidant enzymes (MnSOD and GPX). Reduced production of TNF-α and IL-1β. Decreased accumulation of NF-κB in nucleus, and reduced p53 activation and apoptotic cell death.	[92]
	ECG (5 mg/kg) inhibited the increase of BUN and SCr	Reduced the lipid peroxidation. Restored the activities of renal antioxidant enzymes (SOD and CAT). Increased GSH. Reduced TNF-α and IL-6. Attenuated the activation of MAPK pathway by decrease phosphorylation of ERK1/2, JNK and p38 in renal tissues.	[87]
Contrast-induced nephropathy (CIN)	EGCG (10 mg/kg) normalized SCr and BUN levels, and improved renal histopathological scoring	Reduced the lipid peroxidation. Restored the activities of renal antioxidant enzyme (SOD) and reduced IL-1β via up-regulation of HO-1.	[93]
Ammonium metavanadate-induced AKI (5 mg/kg, i.p.)	EGCG (5 mg/kg) inhibited oxidative stress	Restored the activities of renal antioxidant enzymes (CAT, SOD and GPx). Reduced lipid peroxidation	[88]
**Acute kidney injury induced by obstructive nephropathy**			
Unilateral ureteral obstruction (UUO)	Catechin (2.5, 5 and 10 mg/kg) inhibited oxidative stress	Increased GSH and ROS. Increased mRNA and protein expression of Nrf2 and γ-GCS.	[99]
	EGCG (5 mg/kg) alleviated glomerular and tubular injury and attenuated renal interstitial fibrosis in UUO mice	Decreased macrophage infiltration and reduced production of inflammatory cytokines. Decreased expression of kidney damage markers (KIM-1 and NGAL) via NF-κB and Nrf2 nuclear translocation. Promoted HO-1 production	[96,97]
Renal ischemia-reperfusion injury	Catechin (40 mg/kg) inhibited the increase of BUN and SCr	Reduced lipid peroxidation, increased glutathione levels and restored activities of renal antioxidant enzymes (SOD, CAT and GR).	[89]
	EGCG (50 mg/kg) inhibited the increase of BUN and SCr	Reduced expression of TNF-α, IL-1β, IL-6, Bax and levels of cleaved caspase 3.	[90]
**Acute kidney injury accompanying other morbidities**			
Streptozotocin-induced diabetic nepropathy	Catechins (35 mg/day) prevented functional and morphological deterioration of kidneys, reduced albuminuria and increased creatinine clearance	Catechins reduced lipid peroxidation.	[32,33]
Salt-induced hypertension and renal injury in Dahl salt-sensitive rats (8%)	EGCG (50 mg/kg) reduced of urinary volume, urine protein and renal fibrosis, and increased CCr	Reduced the lipid peroxidation. Decreased the numbers of infiltrating macrophages and T cells. Induced the apoptosis of NRK-49F cells.	[99]
Cardiopulmonary by-pass-induced AKI	EGCG (1 mmol/L) inhibited the increase of SCr	Reduced oxidative stress and kidney damage as demonstrated by lower expression of KIM-1 and less production of 8-hydroxy-20 -deoxyguanosine	[85]

## Data Availability

Not applicable.
